# Ginger (*Zingiber officinale*) phytochemicals—gingerenone-A and shogaol inhibit SaHPPK: molecular docking, molecular dynamics simulations and in vitro approaches

**DOI:** 10.1186/s12941-018-0266-9

**Published:** 2018-04-02

**Authors:** Shailima Rampogu, Ayoung Baek, Rajesh Goud Gajula, Amir Zeb, Rohit S. Bavi, Raj Kumar, Yongseong Kim, Yong Jung Kwon, Keun Woo Lee

**Affiliations:** 1 Division of Applied Life Science (BK21 Plus Program), Systems and Synthetic Agrobiotech Center (SSAC), Plant Molecular Biology and Biotechnology Research Center (PMBBRC), Research Institute of Natural Science (RINS), Gyeongsang National University, Jinju, 52828 Republic of Korea; 2 Primer Biotech Research Center, Jaipuri Colony, Nagole, Hyderabad, Telangana 500068 India; 3 Department of Science Education, Kyungnam University, Changwon, 51767 Republic of Korea; 4 Department of Chemical Engineering, Kangwon National University, Chunchon, 24341 Republic of Korea

**Keywords:** Ginger phytochemicals, 6-Hydroxymethyl-7,8-dihydropterin pyrophosphokinase, GOLD, Shogaol, Gingerenone-A, MD simulations

## Abstract

**Background:**

Antibiotic resistance is a defense mechanism, harbored by pathogens to survive under unfavorable conditions. Among several antibiotic resistant microbial consortium, *Staphylococcus aureus* is one of the most havoc microorganisms. *Staphylococcus aureus* encodes a unique enzyme 6-hydroxymethyl-7,8-dihydropterin pyrophosphokinase (SaHPPK), against which, none of existing antibiotics have been reported.

**Methods:**

Computational approaches have been instrumental in designing and discovering new drugs for several diseases. The present study highlights the impact of ginger phytochemicals on *Staphylococcus aureus* SaHPPK. Herein, we have retrieved eight ginger phytochemicals from published literature and investigated their inhibitory interactions with SaHPPK. To authenticate our work, the investigation proceeds considering the known antibiotics alongside the phytochemicals. Molecular docking was performed employing GOLD and CDOCKER. The compounds with the highest dock score from both the docking programmes were tested for their inhibitory capability in vitro. The binding conformations that were seated within the binding pocket showing strong interactions with the active sites residues rendered by highest dock score were forwarded towards the molecular dynamic (MD) simulation analysis.

**Results:**

Based on molecular dock scores, molecular interaction with catalytic active residues and MD simulations studies, two ginger phytochemicals, gingerenone-A and shogaol have been proposed as candidate inhibitors against *Staphylococcus aureus*. They have demonstrated higher dock scores than the known antibiotics and have represented interactions with the key residues within the active site. Furthermore, these compounds have rendered considerable inhibitory activity when tested in vitro. Additionally, their superiority was corroborated by stable MD results conducted for 100 ns employing GROMACS package.

**Conclusions:**

Finally, we suggest that gingerenone-A and shogaol may either be potential SaHPPK inhibitors or can be used as fundamental platforms for novel SaHPPK inhibitor development.

**Electronic supplementary material:**

The online version of this article (10.1186/s12941-018-0266-9) contains supplementary material, which is available to authorized users.

## Background

*Staphylococcus aureus* has evolved as one of the most devastating pathogens, demonstrating a wide range of antibiotic resistance [1]. *Staphylococcus aureus* is a gram positive, non-motile bacterium. This facultative anaerobe is a gram positive, non-motile bacterium hailing from *Staphylococcaceae* family, powered to infect every known mammalian species causing food poisoning [2, 3]. This is an ectopic commensal and is niched on mucosal membranes and skin of humans [4]. It is transmitted to foods via air, dust, and the lids covering the food containers [5, 6] and the food handlers carry the bacteria on their heads and noses, hence, has an ability to colonize on the normal humans and transmit through direct contact with the bacteria-colonized person. *Staphylococcus* intoxication occurs due to toxin-contaminated food consumption. Such condition is symptomized very quickly (2–8 h) and is associated with vomiting, abdominal cramps, nausea and/or diarrhea [7, 8]. Even though, *staphylococcus* intoxication subsides within 48 h, nevertheless, it becomes severe in children and elders [9] and causes several life threatening infections like, impetigo, ritter disease, osteomyelitis, septic arthritis, endocarditis, toxic shock syndrome, pneumonia, thrombophlebitis and deep skin abscess and infection [10, 11]. Several antibiotics have been used to combat the bacteria [12–20] such as, penicillin, methicillin, oxacillin, various vancomycins and glycopeptides, daptomycin, tetracyclines, aminoglycosides, linezolid, chloramphenicol, florfenciol, macrolides, and streptogramins [21]. However, *Staphylococcus aureus* exerts resistance by several mechanisms that could be broadly categorized into mutations that occur at the chromosomal genes and by horizontally acquired resistance [21]. Specifically, gaining resistance through mutations can happen when the inhibitor is unable to bind to the accurate drug target, derepressing the drug resistance efflux pumps and by mutations that can amend the structure and composition of the drug targets [21]. On the other hand, the horizontally acquired resistance may occur by alteration and inactivation of enzymatic drug, change in the drug binding site, dislocating the drug from its appropriate position and by drug efflux [21]. Adapting either of the mechanisms, the organism endeavours to survive avoiding the encounter with the drug/antibiotic or neutralizes them [22]. Besides these, antibiotic abuse can also add to the raise the resistance [23]. Consequently, the effective treatment is hampered and promotes the infection and enhances the economic burden [23, 24].

Nevertheless, concerns for this bacterium rise due to its resistance against methicillin, often called the Methicillin resistance *S. aureus* (MRSA) that is prevalent currently by exhibiting diverse phenotypes [25]. This ‘superbug’ was responsible for 19,000 deaths in USA in 1 year [26] and can be classified as hospital MRSA (haMRSA), referring to those originating from the hospitals and the community MRSA (caMRSA), indicating to those prevalent in the community [27]. Besides these there is another MRSA called as livestock-associated methicillin resistant *Staphylococcus aureus* [28]. However, caMRSAs are increasingly virulent because of the presence of elevated levels of alpha toxins and phenol-soluble modulin [29]. Not only to methicillin, this bacterium has also been shown resistance against several antibiotics including wonder drug penicillin [1, 22, 30, 31]. Pathogenicity and antibiotic resistance potential of *S. aureus* drives our interest to develop new drugs that can effectively challenge this bacterium.

Choosing an appropriate target for the discovery of novel antimicrobial drugs is a very important aspect [32]. It will be ideal to identify a target that is confined to pathogen and is not present in host such that the drugs can effectively render its efficacy on the target alone, doing no harm to the host [33]. Accordingly, 6-hydroxymethyl-7,8-dihydropterin pyrophosphokinase (HPPK, EC 2.7.6.3) has been chosen as the drug target for the present investigation [33] as it is not present in humans [34]. This monomeric protein comprises of 158 residues, which catalyzes the transfer of pyrophosphoryl group from ATP to 6-hydroxymethyl-7, 8-dihydropterin (HMDP), the substrate. It has a molecular weight of 18 kDa pictured by three-layered α-β-α fold. Typically, the HPPK has three loops comprising of loop 1 with residues from 12 to 14, loop 2 consisting of residues from 45 to 51 and loop 3 consisting of residues from 82 to 94, which demonstrate a significant change in the conformation as compared with the protein structure during catalysis. However, loop 3 is known to display major conformational changes [35]. The rigidity of the ternary structure is attributed when the pterin substrate binds and thus loop 3 closes over the binding site. SaHPPK is an ideal target for novel drug designing due to its expression in pathogen only, and is not targeted by existing antibiotics. Alternatively, this enzyme has also been found in other bacteria such as *Yersinia pestis, Haemophilus influenzae, Streptococcus pneumonia, Francisella tularensis, Saccharomyces cerevisiae* and *Escherichia coli,* demonstrating conserved active sites [36, 37]. Consequently, it can be suggested that the discovery of drugs against SaHPPK might have inhibitory effects on its homologues on other pathogens [36].

An overwhelming significance of phytochemicals to be employed as drug molecules is their capability to induce nutritional values besides acting as a medicine and thus, redirecting towards the formulation of nutraceuticals. Nutraceuticals are the food that have both the nutritional and the pharmaceutical value [38] and refers to as a food or part of the food that induces health benefits while offering medicinal values and thus participates in improving the health of an individual. Additionally, they are cost effective in their formulation and produce no side effects and serious toxicities upon prolonged usage [38]. Due to these properties, the phytochemicals have high advantage to be labelled as drugs [39, 40]. Ginger (*Zingiber officinale*) is an aromatic, pungent and spicy herb, enriched with the natural phytochemicals. For a time, this herbal species has been used as a flavoring agent and has been placed on the top list of folk medicines against common cold [41], sore throat [42] etc. It is therefore understood that ginger is regarded to be safe [39], however, little is known regarding its mechanism of action and hence, careful assessment is required before considering ginger phytochemicals for any therapy [43]. Moreover, ginger extracts were known to showcase its inhibitory effect on *Staphylococcus aureus* [44, 45]. All these intriguing factors focus our research to investigate ginger phytochemicals against SaHPPK and elaborate their mechanism of interaction.

In order to accomplish this goal, we have retrieved eight selected ginger phytochemicals from published literature based upon their therapeutic ability and antimicrobial activity [44, 46–52]. The predictive inhibitory effects of these selected phytochemicals have been evaluated against SaHPPK by molecular docking. To further infer on the mode of binding and interaction with catalytic active residues, successful candidates have been subjected to MD simulations. For the accomplishment of this objective, docking of seven antibiotics, such as streptomycin, ampicillin, amoxicillin, methicillin, penicillin, trimethoprim, and sulfamethoxazole were also considered for comparative analysis. Finally, gingerenone-A and shogaol propounded as potential ginger-phytochemicals against SaHPPK.

## Methods

### Selection of the protein and its preparation

SaHPPK structure used for current investigation, has been obtained from RCSB Protein Data Bank (PDB) with PDB code 3QBC [26], which is in complex with 2-amino-8-sulfanyl-1,9-dihydro-6H-purin-6-one (8MG). The protein was prepared employing Discovery Studio v4.5 (hereinafter D.S v4.5) by removing all heteroatoms and the addition of hydrogen atoms [53]. The structure was energy minimized, until the convergence gradient satisfied was obtained. The active site was evaluated 10.0 Å around 8MG and the key residues were identified as Ala44, Thr43, Val46, and Asn56 [26]. Interrogating the active site revealed the presence of two Phe residues; Phe54 and Phe123, that are located on either side of the 8MG [26]. Moreover, the histidine residues of protein were oriented in accordance with crystal structure to ND1H protonation state.

### Selection and preparation of the ligands

**Fig. 1:**
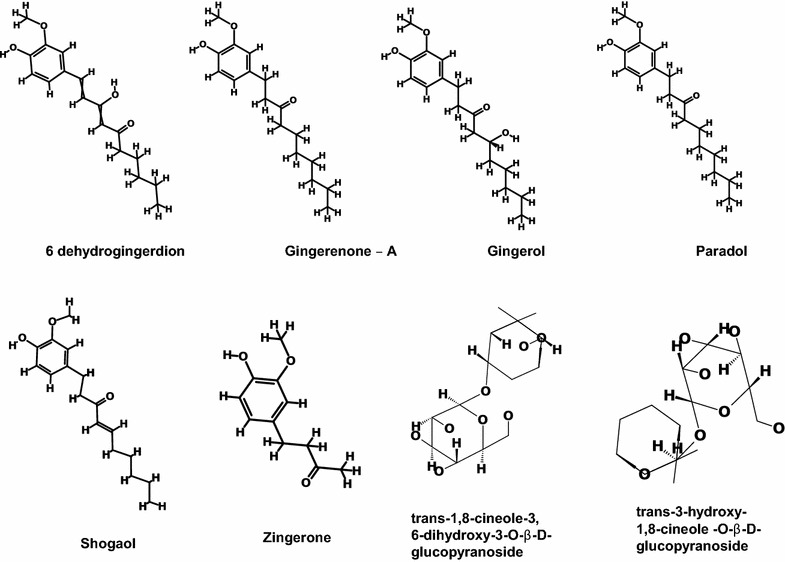
2D structures of the selected phytochemicals

Ginger has several active compounds [54] and to the best of our knowledge, they have not been tested against SaHPPK as no reports were retrieved upon performing a systematic search. This triggered our interest to understand how these phytochemicals effect the SaHPPK and therefore, for the current study, phytochemicals namely 6-dehydrogingerdion, gingerenone-A, gingerol, paradol, shogaol, zingerone, trans-1,8-cineole-3,6-dihydroxy-3-*O*-β-d-glucopyranoside and trans-3-hydroxy-1,8-cineole-*O*-β-d-glucopyranoside were selected that have not been assessed against SaHPPK [54, 55]. More specifically the selection of these phytochemicals was done based upon their therapeutic ability as reported earlier [46–50]. The 2D structures of the selected phytochemicals were represented in (Fig. 1). The corresponding 2D structures of the eight phytochemicals were sketched on ChemSketch (http://www.acdlabs.com/resources/freeware/ChemSketch/) and were subsequently, imported onto D.S v4.5 to generate their 3D structures.

### Molecular docking mechanism

Molecular docking is a promising strategy to mimic intermolecular binding modes and interactions. Particularly, molecular docking relies on binding site topology, intermolecular affinity and interaction of key residues with the ligand. For the current study, Genetic Optimization for Ligand Docking (GOLD) v5.2.2 was employed to perform the docking studies. Goldscore was recruited to compute the binding affinities between the protein and ligands, whereas the Chemscore was used for the rescoring purpose. Goldscore comprises of external H-bond, external vdW, internal vdW and internal torsion. Moreover, to obtain an appropriate binding pattern of ligands, 30 docking poses were allowed to generate. Additionally, for identifying the best pose, the Goldscore, interactions between the protein’s active site residues and ligand and the binding modes were considered.

To further validate the obtained results, a second docking programme, CDOCKER implemented on D.S v4.5 has been employed. The results were evaluated based upon CDOCKER interaction energy; higher CDOCKER interaction energy implies greater favourable binding [56]. This is a grid based docking operates by employing CHARMm and facilities the generation of random ligand conformations retrieved form the initial structure.

### In-vitro antimicrobial analysis of phytochemicals

In-vitro evaluation of the phytochemicals was performed to infer the results obtained from the computational approach. The phytochemicals and the *Staphylococcus aeureus* were the generous gifts from the Osmania University, Department of Botany, Hyderabad. To maintain the prepared medium with no contamination, it was autoclaved for 15 min at 15 lb pressure along with petri dishes, spreader, 4–25 ml conical flasks, forceps, inoculation loops and cotton balls. The agar media was then transferred into the petri dishes and was allowed to solidify.

### Preparation of the culture media

The nutrient agar media was prepared by suspending 28 g of nutrient agar in 1000 ml distilled water according to Mueller and Hinton [57], and was maintained at pH 7.0 and at room temperature.

### Preparation of the inoculum

20 ml of the above-prepared media was transferred onto the petri-dishes and was allowed to solidify. A loopful of bacteria was transferred to 10 ml of distilled water in a test tube and the addition is continued until the turbidity is equal to standard 0.5 McFarland. Employing the cotton swabs the inoculum was gently swabbed on the surface of the media and were then allowed to dry.

### Preparation of the disks

The disks (Whatman 1 filter paper) were prepared with the help of the punch machine of 6 mm in diameter. For the current experiment, the two phytochemicals that have produced the highest dock score along with the antibiotic amoxicillin were considered for current in vitro test. The samples were prepared in the concentration of 1 mg/1 ml.

The bacterial strain, overnight culture was grown in broth was adjusted to an inoculum size of 106 CFU/ml for inoculation of the agar plates. The sterilized disks were carefully impregnated with the sample, allowed to dry for 1 min, and then transferred onto the petri dishes containing 20 ml on nutrient agar. The plates were allowed to incubate for 24 h at 35 ± 2 °C and was followed by measuring the zone of inhibition expressed in mm and was performed in triplicates [58].

### Minimum inhibitory concentration

To quantify the minimum inhibitory concentration (MIC), different concentration of the phytochemicals were tested against the *Staphylococcus aureus* (MTCCB 737) ranging between 0.05 and 2 mg/ml. The MIC is defined as the lowest concentration of the phytochemical (highest dilution) that can inhibit the growth of the bacteria.

### Statistical analysis

In vitro results were analyzed employing the GraphPad Prism v7.02 and were expressed as mean ± standard deviation recruiting the Turkey’s method and the correlation analysis was executed by two-way ANOVA, *P value* less than 0.005 was considered as significant.

### Molecular dynamics simulations to assess the binding modes of the hits against the reference

To gain further insight into the protein–ligand interactions, the selected ginger phytochemicals were subjected to MD simulations along with reference compound (8MG) and amoxicillin. Parameters for protein’s topology and coordinates were developed by CHARMm27 ff [59–62] in GROMACS 5.0.7 [63]. The topologies of the ligands and the cofactor were extracted from the SwissParam [64]. The parameters for topology and coordinates of protein and for corresponding ligand were merged, and ten independent systems (one for each phytochemical, one for amoxicillin, and one for 8MG) were designed. Each system was solvated in a dodecahedron box, using TIP3P water model, and neutralized with counter ions. Each solvated system was energy minimized by employing steepest descent algorithm for 10,000 steps and an upper limit of force being lower than 1000 kJ/mol was employed to remove any bad contacts and steric clashes of protein–ligand complexes. Every minimized system was grouped into protein–ligand and solvent-ions to escape collapse, and subsequently, subjected to equilibration. The equilibration of each system was comprised of two components. First, equilibration was conducted at constant volume (NVT) for 1 ns at constant temperature of 300 K using Berendsen thermostat algorithm [65]. Following this, second equilibration was executed for 1 ns at constant pressure (NPT) of 1 bar maintained by Parrinello–Rahman barostat [66] and LINCS [67] was employed to constrain all bonds. Particle Mesh Ewald (PME) [68] was used to calculate long-range electrostatic interactions with a cut-off of 1.2 nm. All short-range non-bonded interactions were calculated within a cut-off of 1.2 nm. A cut-off distance of 12 Å was attributed for Coulombic and van der Waals interactions. All simulations were executed using the NPT ensemble for 100 ns, and coordinates were saved after each 2 fs intervals. The results were examined recruiting visual molecular dynamics [69] and D.S v4.5.

## Results

### Molecular docking

Molecular docking results showed that the eight ginger phytochemicals have strong interactions with SaHPPK protein, however, gingerenone-A and shogaol displayed the highest Goldscore of 63.62 and 55.48 respectively (Additional file 1: Table S1). On the other hand, it was noted that the antibiotics showed lower Goldscore than the phytochemicals. Among them, amoxicillin has generated the highest dock score of 41.98 (Additional file 1: Table S2), and therefore, this antibiotic was considered for further studies. Hereinafter, the co-crystal, 8MG in SaHPPK crystal structure, was designated as the reference compound. Characteristically, this is a co-crystal located at the active site of the protein and imparts knowledge on the location where the chosen ligands should be anchored at the proteins binding groove. Additionally, this guides the key residues that are involved in the inhibition and marked at 10.0 Å around its location. Moreover, an interaction with these residues labels prospective drug candidates as effective. Furthermore, the reference molecule should logically determine an appropriate binding mode of the candidate molecules. Therefore, the 8MG has been represented as the reference compound. To further evaluate the best conformation, the dock scores rendered by GOLD and CDOCKER, catalytic active residue(s) interactions and the binding modes were opted as the determinant factors. The study was proceeded in the presence of Mg^2+^-AMPCC.

### In-vitro antimicrobial analysis of phytochemicals

**Fig. 2:**
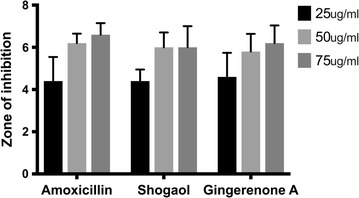
Antimicrobial activity of shogaol, gingerenone-A and amoxicillin expressed by zone of inhibition in mm

The ginger phytochemicals have rendered remarkable results and were comparable with the standard reference antibiotic and a control into which no inoculum was added. The mean zone of inhibition of the phytochemical shogaol has recorded to be between 6 and 12 mm and gingerenone-A was found to be between 2 and 8 mm, respectively. These reading are in harmony with that of the reference antibiotic and were observed to be 4–16 mm. The minimum zone of inhibition was observed at 25 µg/ml for the phytochemicals and the reference antibiotic. The in vitro results further state that the phytochemicals could induce the inhibitory effect in par with amoxicillin (Fig. 2).

### Molecular dynamics simulations

**Fig. 3:**
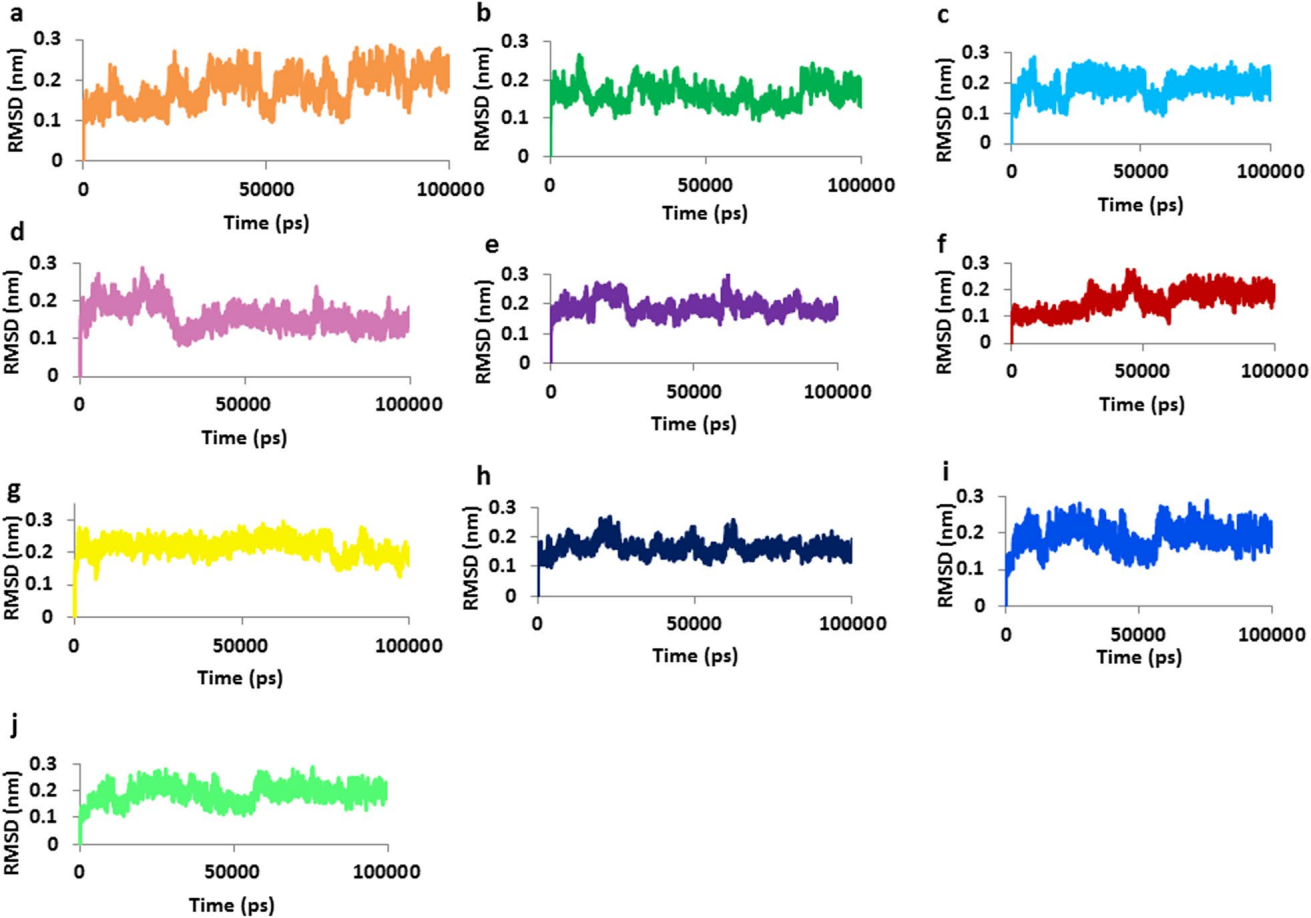
RMSD profiles of ten systems during 100 ns. The plots show variations during initial simulations and are stable towards last 20 ns. **a** Reference, **b** amoxicillin, **c** gingerenone-A, **d** gingerol, **e** shogaol, **f** zingerone, **g** 6dehydrogingerdion, **h** paradol, **i** trans-1,8-cineole-3,6-dihydroxy-3-*O*-β-d-glucopyranoside, **j** trans-3-hydroxy-1,8-cineole-*O*-β-d-glucopyranoside

**Fig. 4:**
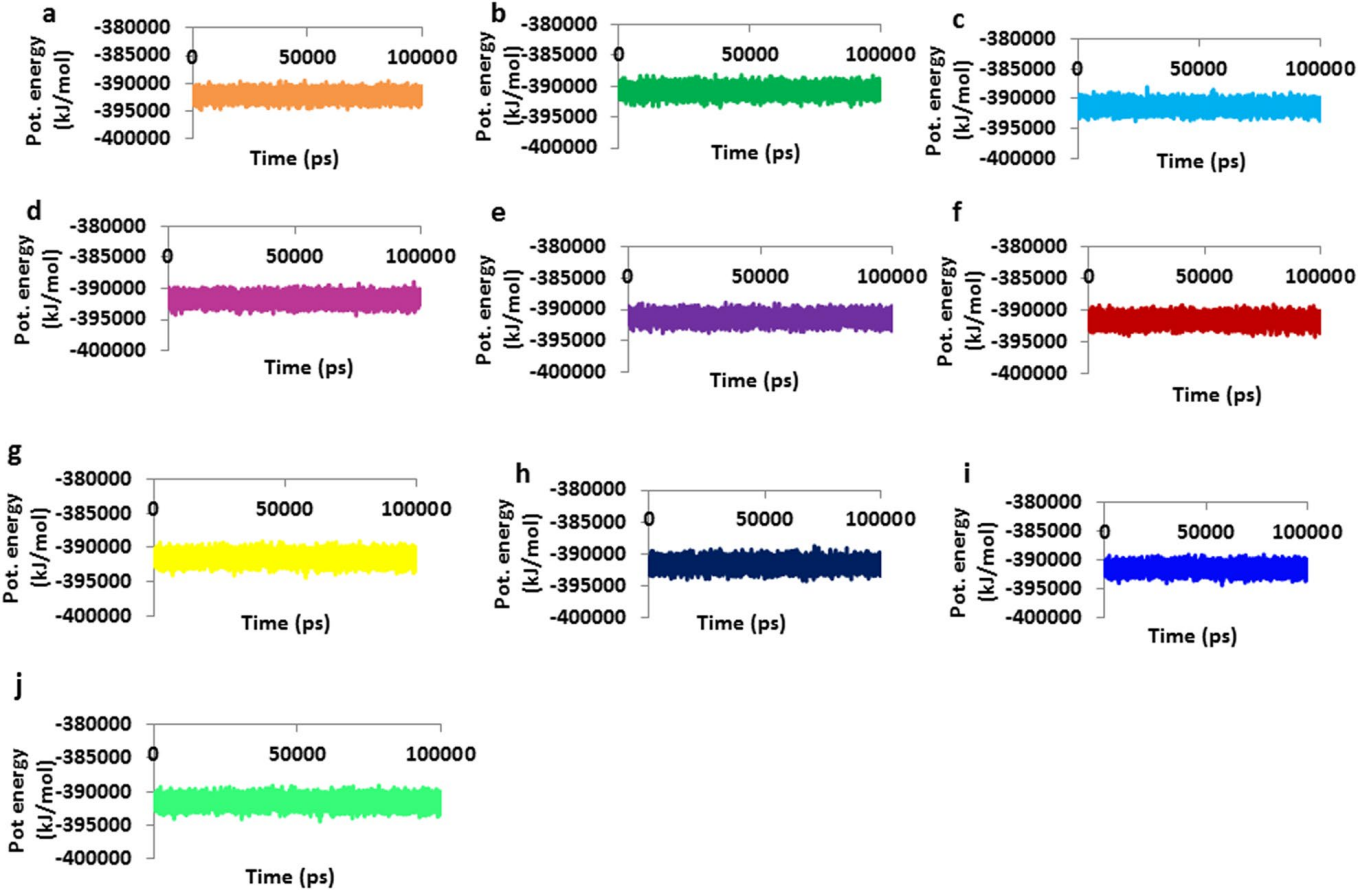
Potential energy plots of ten systems during 100 ns. The plots appear to be well converged between − 390,000 and − 395,000 kJ/mol. **a** Reference, **b** amoxicillin, **c** gingerenone-A, **d** gingerol, **e** shogaol, **f** zingerone, **g** 6dehydrogingerdion, **h** paradol, **i** trans-1,8-cineole-3,6-dihydroxy-3-*O*-β-d-glucopyranoside, **j** trans-3-hydroxy-1,8-cineole-*O*-β-d-glucopyranoside

**Fig. 5:**
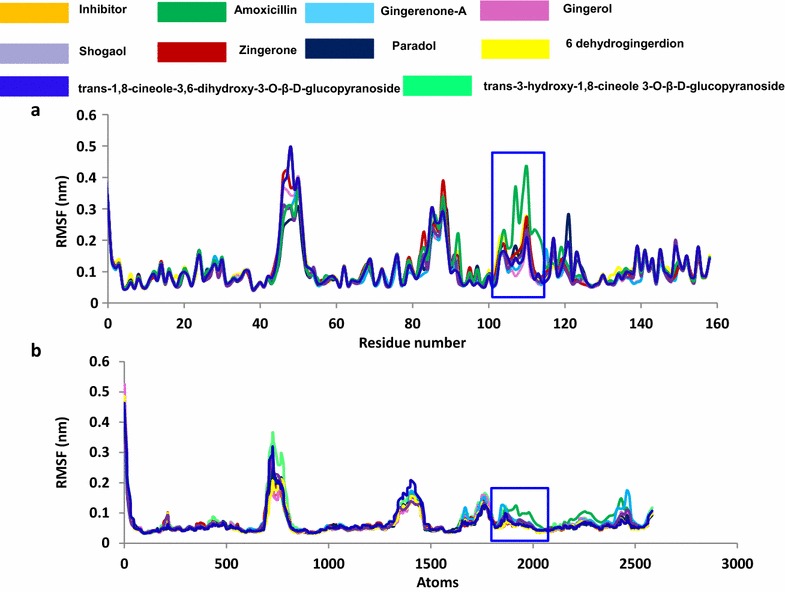
RMSF plots during 100 ns. Blue box denotes the variations notices in the profiles. The RMSF profile of amoxicillin is found to be relatively deviated. **a** The RMSF of the residues. **b** The RMSF of the corresponding fluctuating atoms

To gain insight into the interaction mechanism of ligands (selected phytochemicals, amoxicillin, and reference compound), MD simulations were carried out [70–72] for 100 ns and the results were analyzed. Throughout the MD run, dynamic behavior and conformational changes of all the ligands were monitored. The root mean square deviation (RMSD) of backbone atoms was evaluated to estimate the stability of each protein–ligand complex. The reference molecule displayed an average RMSD of ~ 0.18 nm, while gingerenone-A and shogaol projected ~ 0.15 and ~ 0.16 nm respectively (Fig. 3), while amoxicillin has demonstrated a RMSD of ~ 0.17 nm. Additionally, all RMSD plots were noticed to be in 0.1–0.27 nm range (Additional file 1: Figure S1) suggesting their stability during the simulation [73]. Furthermore, the potential energy analysis notified that all the compounds have ranged between − 390,000 and − 395,000 kJ/mol, (Fig. 4). The root mean square fluctuation (RMSF) profiles of backbone atoms of the corresponding systems shed light on their fluctuations. Accordingly, for all the systems, backbone fluctuation was observed within 0.49 nm and was found to be in similar manner. However, moderate deviations were noticed for amoxicillin between 0.1 and 0.4 nm as depicted in blue box with reference to the residues that lie between 90 and 110 (Fig. 5a) and their corresponding atoms (Fig. 5b). This deviation might be because of the non-bonded water molecule that is present in the active site.

**Fig. 6:**
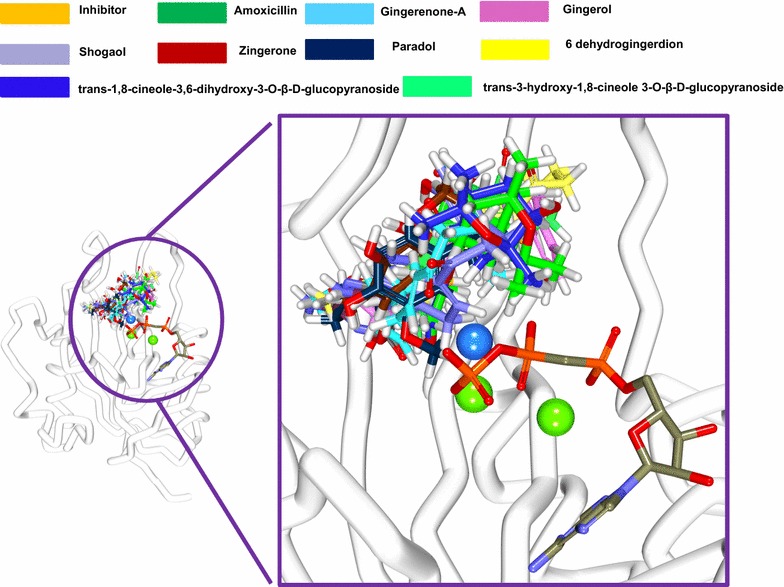
Binding pattern of the co-crystal and the ginger phytochemicals. Only polar carbons are shown for clarity. Figure on the left depicts the superimposition of the ligands and figure right is its enlarged structure. The protein is represented in steel and the ligands in stick. The water molecule is denoted in blue and the Mg^2+^ ions in green

**Fig. 7:**
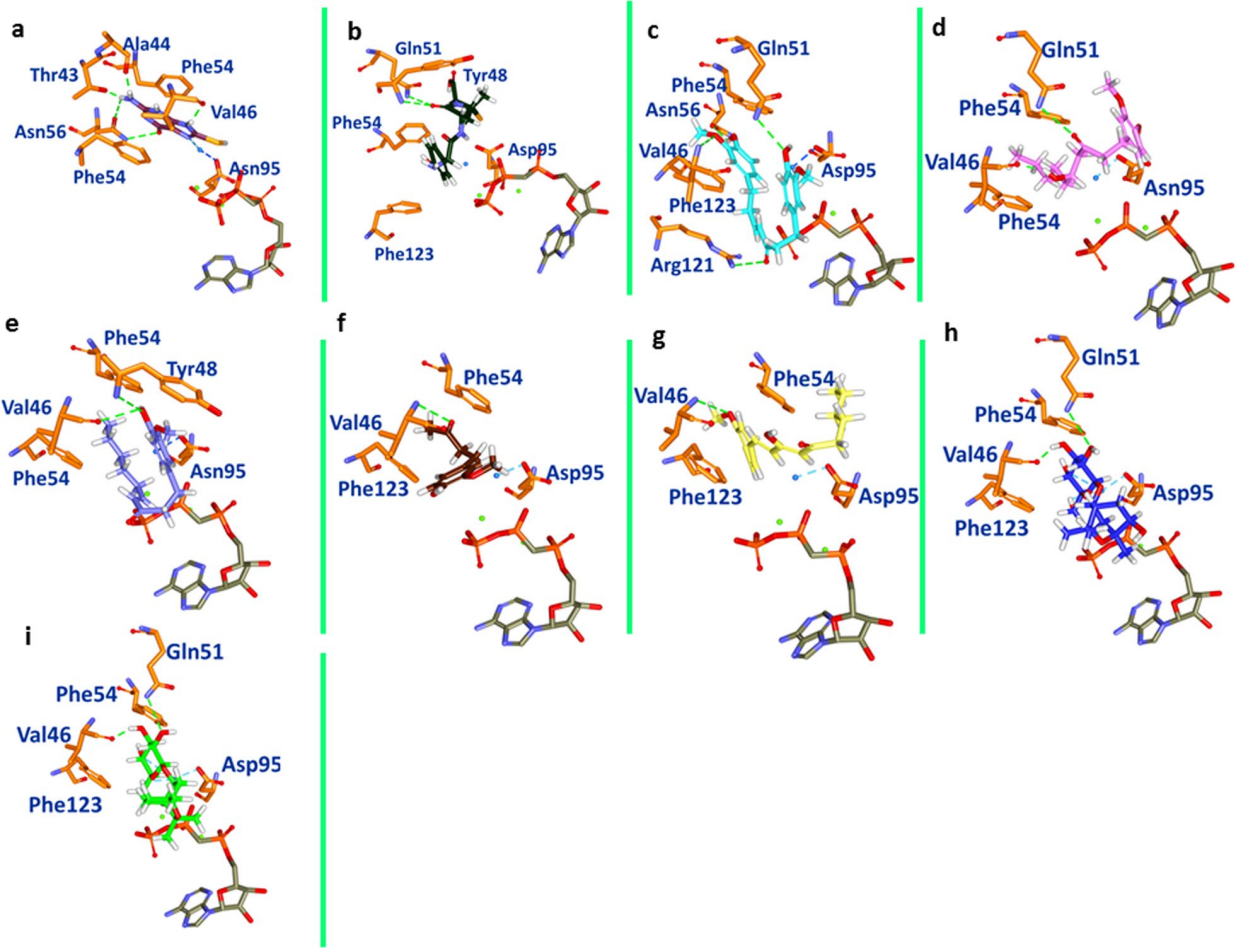
Molecular interactions and the binding mode conformation of the reference and the phytochemicals with the protein target. Green dashed lines demonstrate the hydrogen bonds between the protein and the ligands. The blue dashed lines represent the binding of the water molecule and Asp95. The protein is represented in orange stick. The water molecule is denoted in blue and the Mg^2+^ ions in green. **a** Reference, **b** amoxicillin, **c** gingerenone-A, **d** gingerol, **e** shogaol, **f** zingerone, **g** 6dehydrogingerdion, **h** paradol, **i** trans-1,8-cineole-3,6-dihydroxy-3-*O*-β-d-glucopyranoside, **j** trans-3-hydroxy-1,8-cineole-*O*-β-d-glucopyranoside

The binding mode assessment was executed utilizing the last 20 ns trajectories. Upon superimposition of the representative structure of each system, it was observed that all the ligands have occupied the binding pocket in a similar manner as was observed for reference compound (Fig. 6) and displayed similar binding pattern. The binding pocket is present towards the loop 2 and the cofactor site is located near to loop 3. Since the target protein is devoid of cofactor AMPCC and two Mg^2+^ ions, we have imported their coordinates from SaHPPK protein with the PDB code 5ETR. Additionally, the key residues that shaped the active site were found to interact with the reference compound as well as for the selected phytochemicals. Inspecting the molecular interactions revealed that the reference compound has generated four hydrogen bonds with key residues of SaHPPK (Fig. 7a). The carboxylic oxygen (hereinafter O) of Ala44 has interacted with N3 of reference compound, whereas, O of Val46 has H-bond interaction with N5 of reference compound. One H-bond was detected between the OG1 of Thr43 residue and N3 of reference compound, while another H-bond was formed between ND2 of Asn56 residue and O1 of reference compound. OD1 atom of Asn56 additionally participated in H-bond interaction with the H13 atom of the ligand. Furthermore, it was observed, that all the H-bonds displayed a distance of ~ 2.8 Å.

Amoxicillin formed one hydrogen bond between N atom of Tyr48 has formed the H-bond with the O2 atom of the ligand with a distance of 2.9 Å, while another H-bond was observed for NE22 of Gln51 and O2 of amoxicillin with a bond distance of 2.9 Å (Fig. 7b).

Hydrogen bond interactions with key residues were noticed with all the phytochemicals, however their number varied significantly (Fig. 7). To further authenticate our results, we have done a comparison with the co-crystal inhibitor and the known antibiotic. The active site and the key residues (Ala44, Thr43, Val46, and Asn56) were defined 10.0 Å around the inhibitor. The prospective drug molecules were examined critically for their interactions with these residues. Amongst them, phytochemicals, shogaol, gingerol, trans-1,8-cineole-3,6-dihydroxy-3-*O*-β-d-glucopyranoside and trans-1,8-cineole-3,6-dihydroxy-3-*O*-β-d-glucopyranoside have displayed 2 H-bonds with protein, 6-dehydrogingerdione and zingerone have formed one H-bond each while gingerenone-A displayed 4 H-bonds (Fig. 7).

Further delineating, it was noted that one H-bond was observed between the N of Val46 and O2 of gingerenone-A with a bond distance of 2.3 Å. The NE2 of Gln51 has interacted with the O4 of the ligand with a distance of 2.9 Å. Another H-bond was noticed between the H44 of the gingerenone-A and OD1 of Asn56 with a distance of 2.0 Å, while the other H-bond was detected between NH2 of Arg121 and O3 of gingerenone-A with bond distance of 1.3 Å, (Fig. 7c). Gingerenone-A also formed water-mediated interaction with Asp95 of SaHPPK. On the other hand, gingerol formed two hydrogen bonds with Val46 and Gln51 each. The O atom of the Val46 has interacted with H37 of gingerol with a distance of 1.9 Å. Gln51 has participated in the H-bond formation with its NE2 atom and the O2 of the ligand with a distance of 2.9 Å (Fig. 7d). Moreover, shogaol formed two H-bond with Val46 and Tyr48, respectively. The H41 atom of the ligand has formed a bond with the O of Val46 with a distance of 2.9 Å, while the N atom of Tyr48 has interacted with the O3 of the ligand with a distance of 2.9 Å (Fig. 7e). Additionally, a water-mediated bond with Asp95 stabilized shogaol. Zingerone has also generated an H-bond between N of Val46 and O3 of ligand with a bond distance of 2.9 Å, (Fig. 7f). When 6-dehydrogingerdion was evaluated for H-bond analysis, it was observed that O3 of the inhibitor and N of Val46 formed a single H-bond with a bond length of 2.5 Å, (Fig. 7g). Phytochemical paradol did not render any hydrogen bond; however, it had shown van der Waals interactions Table 1. Phytochemical trans-1,8-cineole-3,6-dihydroxy-3-*O*-β-d-glucopyranoside demonstrated two H-bonds with Val46 and Gln51. The O atom of Val46 has involved in the hydrogen bond with H48 of the ligand with a distance of ~ 1.8 Å. The second bond was formed between the HE22 of Gln51 and O20 of the ligand represented by a bond distance of 2.1 Å, (Fig. 7h). The compound trans-3-hydroxy-1,8-cineole-*O*-β-d-glucopyranoside has rendered 2 H-bonds anchored by Val46 and Gln51, respectively. The O atom of Val46 and H47 of the ligand joined by a H-bond displaying a bond length of 1.8 Å. The second H-bond was formed between the HE22 atom of Gln51 and O19 of the ligand with a distance of 2.1 Å, (Fig. 7i).

**Table 1:** Molecular interactions between the protein and the compound

Compound	H-bond (< 3.0 Å)	van der Waals interactions	π-Alkyl
Inhibitor	Ala44, Val46, Thr43, Asn56	Gly9, Pro45	Val46
Amoxicillin	Tyr48, Gln51	Gly9, Thr93, Asn11, Ile12, Thr43, Pro45, Gly47, Tyr48, Thr93	Leu57
Gingerenone-A	Val46, Gln51, Asn56, Arg121	Gly9, Ala44, Gly47, Pro45, Phe54, Asn56, Asp97, His115, Glu12, Phe149, Val154, Asp151, Ser153	Ala122
Gingerol	Val46, Gln51	Ala44, Asn56, Asp95, Arg121, Ala122, Pro127, Ser153	Phe123, Val154
Shogaol	Val46, Tyr48	Gly7, Leu8, Gly9, Thr43, Ala44, Asn56, Phe54, Val96, Asp95, Asp97, Leu99, Glu120, Ala122, Phe149, Val154, Asp151, Ser153, His115	Val46, Arg121, Val124, Phe 123
Zingerone	Val46	Gly7, Ser10, Asp95, Val96, Arg121	Val46
6-Dehydrogingerdion	Val46	Leu8, Ser10, Ala44, Asn56, Arg121, Arg151	Ala122
Paradol	–	Gly7, Leu8, Gly9, Thr43, Ala44, Pro45, Asn56, His115, Glu120, Val124, Asp151, Ser153, Val154	Val46, Ala122
Trans-1,8-cineole-3,6-dihydroxy-3-*O*-β-d-glucopyranoside	Val46, Gln51	Pro45, Gly47, Tyr48, Phe54, Phe123	–
Trans-3-hydroxy-1,8-cineole-*O*-β-d-glucopyranoside	Val46, Gln51	Pro45, Gly47, Tyr48, Phe54, Phe123	–

Focusing on the importance of water molecule in SaHPPK stability and augmenting its reactivity, it was speculated that water molecule at active site plays a crucial rule (Fig. 7). The water mediated bond with Asp95 was noticed in the presence of all the phytochemicals as was observed with the reference molecule, however this interaction was absent with the antibiotic amoxicillin, (Fig. 7b). Further details of the interactions are recorded in Table 1. The resultant docked poses were validated by MD simulation analysis and it was confirmed that the binding stability of the selected poses remained unaltered during the simulation.

The interactions of the cofactor and the Mg^2+^ with the protein were additionally evaluated that the benzene ring of the adenine group has interacted with three hydrogen bonds formed by Ile98 and Ser112 residues. Leu71 additionally holds the adenine group by the hydrophobic interactions. The ribose moiety has interacted with Lys110 demonstrated by a hydrogen bond. Arg121 has interacted with O1G of the cofactor by electrostatic bond. Furthermore, the electrostatic bonds hold the Mg^2+^ ions represented by Glu78 and Asp97. Additionally, the exposed O atoms of the cofactor firmly hold the Mg^2+^ ions (Additional file 1: Figure S2).

## Discussion

Despite tremendous progress in medical sciences, the effective treatment against infectious microorganisms remains a major challenge. The primary reason behind this failure is the ability of the microorganisms to gain resistance against antibiotics, which is conferred by a variety of mechanisms. Consequently, it is essential to develop new drugs that can effectively combat the microorganisms and to overcome their pathogenicity. *Staphylococcus aureus* is one of the widely known pathogenic microorganisms that has gained resistance against several antibiotics. In this study, ginger phytochemicals were employed to evaluate their inhibitory effects when challenged against microbial pathogenicity. Since, HPPK plays a key role in microbial folate pathway and hence, SaHPPK might be an ideal target for novel inhibitors. Additionally, an ideal target should possess the following attributes, such as having no homolog in humans, should exist in large range of bacteria performing characteristics role, should be specifically druggable and should possess a low cross-resistance potential (https://www.ncbi.nlm.nih.gov/books/NBK200811/#sec_17). Because SaHPPK represents all these characteristic features, we have relied on SaHPPK for our current investigation.

Besides, the selection of the phytochemicals have been performed based upon the literature search taking into consideration that the phytochemicals portray and are embedded with therapeutic activities [46–50]. We aimed at understanding how these compounds that have a similar structure act when challenged against SaHPPK and further which phytochemical is potential against the targeted protein. Such a study was conducted earlier considering different phytochemicals against different targets and diseases [74–77]. The study was conducted in the presence of Mg^2+^-AMPCC to determine the effect of the chosen phytochemicals.

Our investigation of eight ginger phytochemicals against SaHPPK demonstrated that two candidate phytochemicals gingerenone-A and shogaol have higher inhibitory effects. Furthermore, highest dock score, stable orientation of selected phytochemicals in SaHPPK active site and stable interactions with key residues, support our investigation. Furthermore, to validate the dock results, we have performed the in silico investigation employing second docking tool, CDOCKER. These docking results reaffirm the superiority of gingerenone-A and shogaol rendered by highest CDOCKER interaction energy (Additional file 1: Table S1) and the interaction with key residues located at the active site of the protein. Additionally, different interactions also affirmed that gingerenone-A and shogaol might be potential scaffolds to be developed has novel SaHPPK inhibitors.

In the current study, all the phytochemical have displayed a score greater than the reference, however, amongst them, since, gingerenone-A and shogaol projected remarkable scores by both the molecular docking programmes and further displayed stable MD results, we therefore have considered only the top two dock scored compounds for further evaluation and subsequently for the in vitro experiments. Delineating on in vitro results, it can be observed that the lead candidates have exhibited remarkable inhibition at all the concentrations including at 25 µg/ml. However shogaol has demonstrated an overall greater inhibition at 50 µg/ml while gingerenone-A rendered a marginally higher degree of inhibition at 75 µg/ml. Nevertheless, both the phytochemicals have conferred with the inhibitory activities in par with the antibiotic thus; affirm the inhibitory potential of ginger phytochemicals against SaHPPK.

Since, it is widely accepted that *S. aureus* is resistant to existing antibiotics; we speculate that none of these antibiotics have potential to inhibit SaHPPK. One of the possible reasons for this failure is the missing of H-bonding of amoxicillin with Val46. Valine at position 46 of SaHPPK has been reported as key catalytic residue and forms stable H-bond with reference compound [26]. Our findings showed that the phytochemicals as well as the reference compound formed stable H-bond with Val46 after 100 ns MD simulation, while such a binding pattern was not observed for amoxicillin. Delineation on Val46 and its significance, we scrupulously monitored the interaction with their respective atoms of the ligands throughout the MD run. Val46 was seen to anchor with the ligands within the acceptable hydrogen bond length of < 3 Å. Conversely, amoxicillin failed to represent the substantial interaction. This comparison and MD examination led us to the conclusion that Val46 is crucial in any SaHPPK targeted small molecule therapy. Apart from that, water molecule plays very important role in increasing the accuracy and feasibility of chemical reactions [78, 79]. The co-crystal structure of SaHPPK also proclaimed a crucial role of the water molecule [26]. SaHPPK crystal structure revealed that the reference compound is stabilized in enzyme’s active site by a water-mediated bond present at Asp95 of the enzyme. Our results also showed the same pattern of water-mediated stability for all phytochemicals as well as the reference compound (Fig. 7). Conversely, amoxicillin could not gain water-mediated stability in SaHPPK active site. Based on our results we emphasize that the interaction of Val46 residue and water-mediated interaction of Asp95 are the pre-requisite for SaHPPK functional exploration and/or inhibition.

The crystal structure of protein SaHPPK PBD code: 3QBC, has three loops loop 1, loop 2, loop 3; comprising of 12–14, 45–51 and 82–94 residues, respectively [26]. As in the crystal structure, the co-crystal substrate 8MG has been sandwiched between aromatic residues Phe54 and Phe123. Our results also confirmed similar π–π stacked interactions between the ligand molecules and the Phe54 and Phe123 of the crystal structure.

**Fig. 8:**
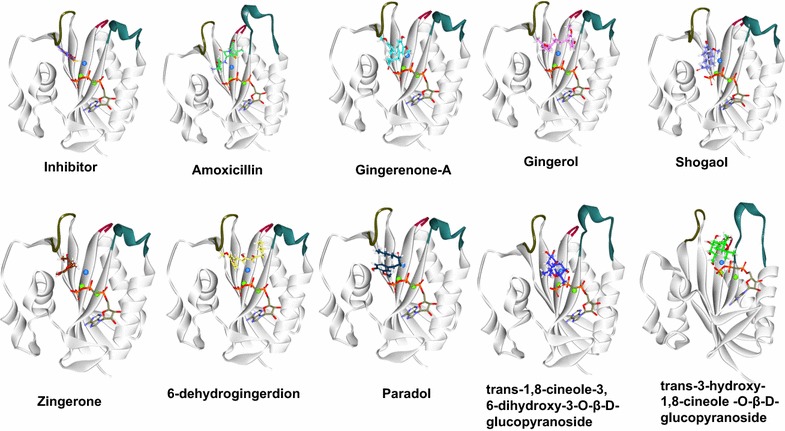
Different conformations exhibited by loop 1 (residues 1–9, in brown loop 2 (residues 43–53, denoted in olive green) and loop 3 (residues 82–92, represented in bottle green). Loop 1 and loop 2 remained semi-closed and closed in all the complexes, while the conformational changes were noticed with loop 3. The protein is represented in steel and the ligands in stick. The water molecule is denoted in blue and the Mg^2+^ ions in green

We further investigated the structural topology and the behaviour of the three loops upon the interaction with the phytochemicals and the evaluation was based upon the findings of Kaifu et al. [80]. Loop 1 showed the semi-open conformation, while loop 2 and loop 3 demonstrated the closed conformations as represented in (Fig. 8 and Table 2). From the results, it was evident that loop 2 predominantly displayed closed conformation and loop 3 on the other hand showed closed conformation except for amoxicillin. These results further impart information that the presence of AMPCC in the cofactor site has induced the closed conformation of loop 3. We further speculated that Asp95 that lies in close proximity with loop 3 might also have played a role in providing closed conformation while it shows semi-closed conformation with amoxicillin, in which case the Asp95 binding was absent (Fig. 8).

**Table 2:** Table depicting different conformational changes of the loops

Compound name	Loop 1	Loop 2	Loop 3
Inhibitor	Semi closed	Closed	Closed
6-Dehydrogingerdione	Semi closed	Closed	Closed
Gingerol	Semi closed	Closed	Closed
Zingerone	Semi closed	Closed	Closed
Amoxicillin	Semi closed	Closed	Semi-closed
Shogaol	Semi closed	Closed	Closed
Gingerenone-A	Semi closed	Closed	Closed
Paradol	Semi closed	Closed	Closed
Trans-1,8-cineole-3,6-dihydroxy-3-*O*-β-d-glucopyranoside	Semi closed	Closed	Closed
Trans-3-hydroxy-1,8-cineole-*O*-β-d-glucopyranoside	Semi closed	Closed	Closed

We further assessed to comprehend the binding ability of the phytochemicals across different homologues. It is well reported that the HPPK structures are highly conserved with six-strands in α-β-α fold with Mg^2+^ ions that recognize the ATP and HMDP substrates. Additionally, among the present HPPK proteins, the *S. aureus* homologue shares the identities with *E. coli* and *Y. pestis* by 39%, *H. influenza* and *S. cerevisiae* by 37 and 34% with *S. pneumonia* [36, 81]. It can be speculated that, since the organisms share a conserved active site and high structural similarity of the ternary complexes, the identified phytochemicals could also exert their antimicrobial effect on its homologues as was reported earlier [36].

Based upon the results obtained from molecular docking, MD simulation and in vitro studies it can be indicated that gingerenone-A and shogaol might be more effective against SaHPPK. Consequently, we suggest these two ginger-phytochemicals as foundation scaffolds for the development of novel SaHPPK inhibitors.

## Conclusion

The best way to increase the antibiotic activity especially for the multidrug resistant bacteria is the inclusion of natural products into the drug formulation, as they offer a plethora of advantages. In the present investigation, ginger phytochemicals were evaluated for the prospective drugs. Out of the eight phytochemicals, shogaol, and gingerenone-A were potential drug candidates demonstrating highest dock scores and strong active site residue interactions. Furthermore, the MD simulation results confirmed their stable orientation and strong interactions with catalytic active residues of SaHPPK catalytic pocket. We therefore speculate that the two phytochemicals can be effective against SaHPPK.

## Additional file

**Additional file 1: Table S1.** Ginger phytochemicals with their respective dock scores. **Table S2.** Antibiotics and their dock score. **Figure S1.** RMSD cluster of eight systems. **Figure S2.** Interaction of cofactor and Mg^2+^ with the protein.
