# Supplementary material for: Ginger (Zingiber officinale) phytochemicals—gingerenone-A and shogaol inhibit SaHPPK: molecular docking, molecular dynamics simulations and in vitro approaches
Source: Ann Clin Microbiol Antimicrob. 2018 Apr 2;17:16. doi: 10.1186/s12941-018-0266-9 (PMC5879566; doi:10.1186/s12941-018-0266-9)
Supplement: Supplementary file 1 — Additional file 1: Table S1. Ginger phytochemicals with their respective dock scores. Table S2. Antibiotics and their dock score. Figure S1. RMSD cluster of eight systems. Figure S2. Interaction of cofactor and Mg2+ with the protein. [file 12941_2018_266_MOESM1_ESM.pptx]

## Slide 1
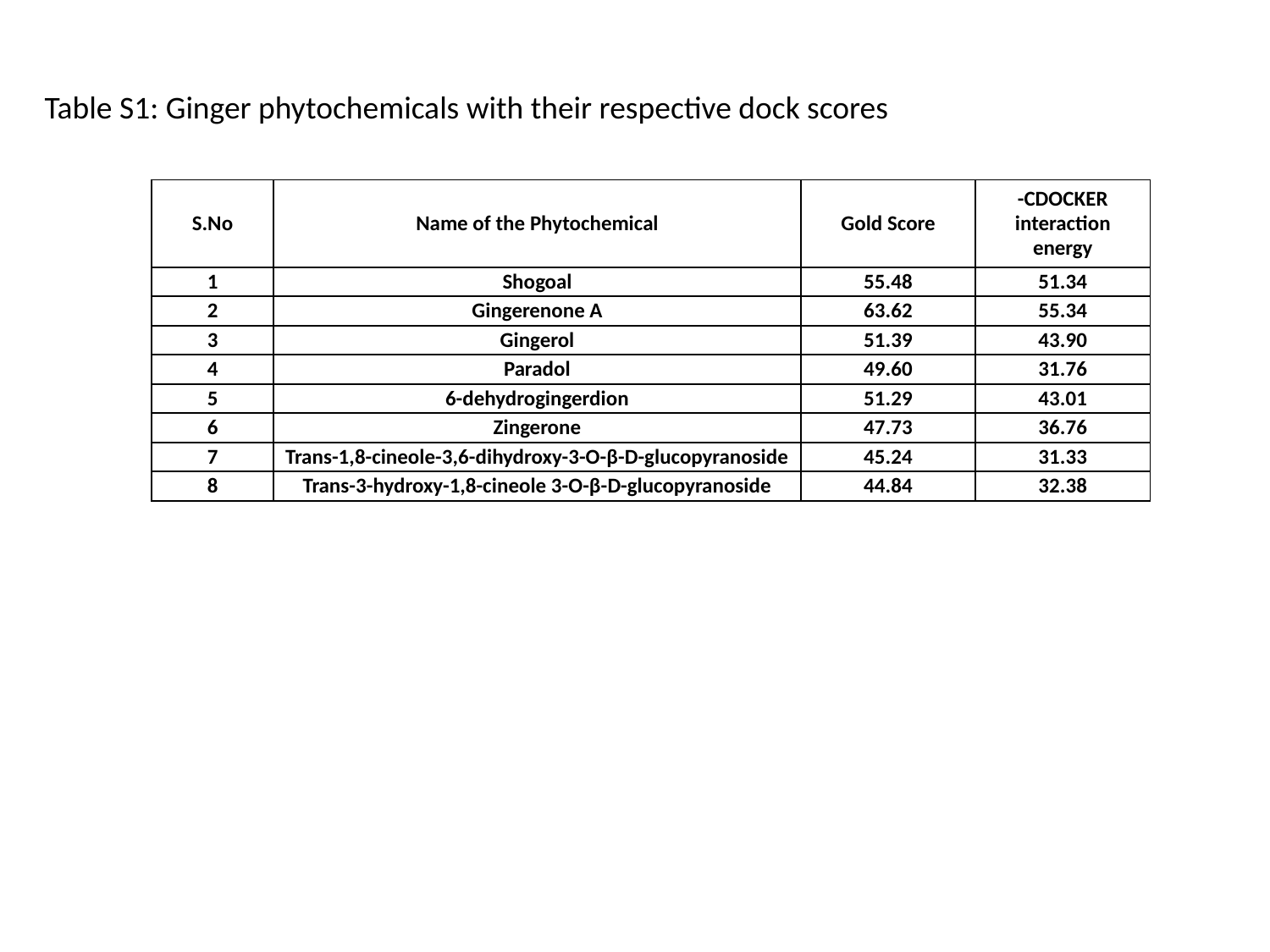

Table S1: Ginger phytochemicals with their respective dock scores
| S.No | Name of the Phytochemical | Gold Score | -CDOCKER interaction energy |
| --- | --- | --- | --- |
| 1 | Shogoal | 55.48 | 51.34 |
| 2 | Gingerenone A | 63.62 | 55.34 |
| 3 | Gingerol | 51.39 | 43.90 |
| 4 | Paradol | 49.60 | 31.76 |
| 5 | 6-dehydrogingerdion | 51.29 | 43.01 |
| 6 | Zingerone | 47.73 | 36.76 |
| 7 | Trans-1,8-cineole-3,6-dihydroxy-3-O-β-D-glucopyranoside | 45.24 | 31.33 |
| 8 | Trans-3-hydroxy-1,8-cineole 3-O-β-D-glucopyranoside | 44.84 | 32.38 |

## Slide 2
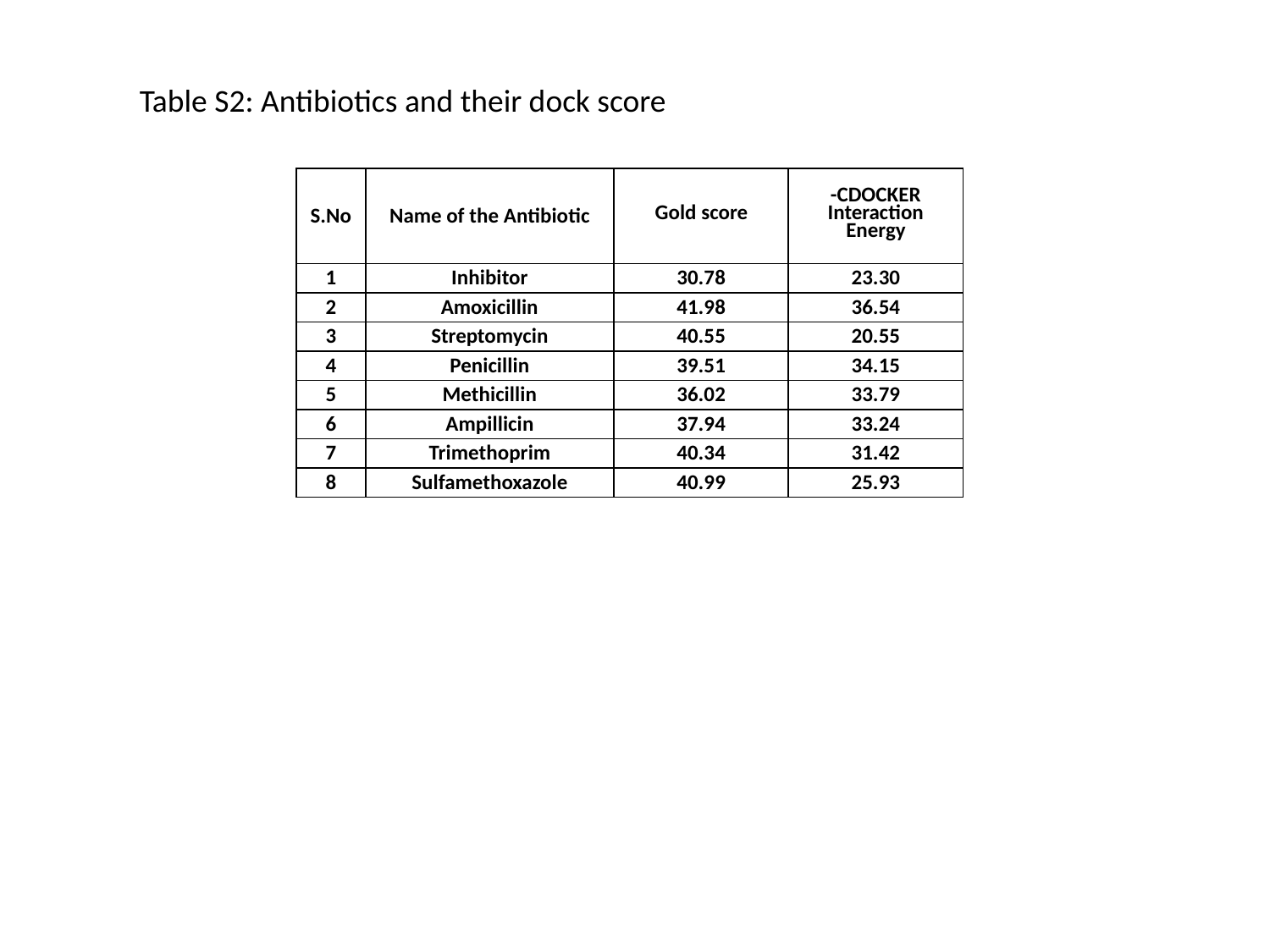

Table S2: Antibiotics and their dock score
| S.No | Name of the Antibiotic | Gold score | -CDOCKER Interaction Energy |
| --- | --- | --- | --- |
| 1 | Inhibitor | 30.78 | 23.30 |
| 2 | Amoxicillin | 41.98 | 36.54 |
| 3 | Streptomycin | 40.55 | 20.55 |
| 4 | Penicillin | 39.51 | 34.15 |
| 5 | Methicillin | 36.02 | 33.79 |
| 6 | Ampillicin | 37.94 | 33.24 |
| 7 | Trimethoprim | 40.34 | 31.42 |
| 8 | Sulfamethoxazole | 40.99 | 25.93 |

## Slide 3
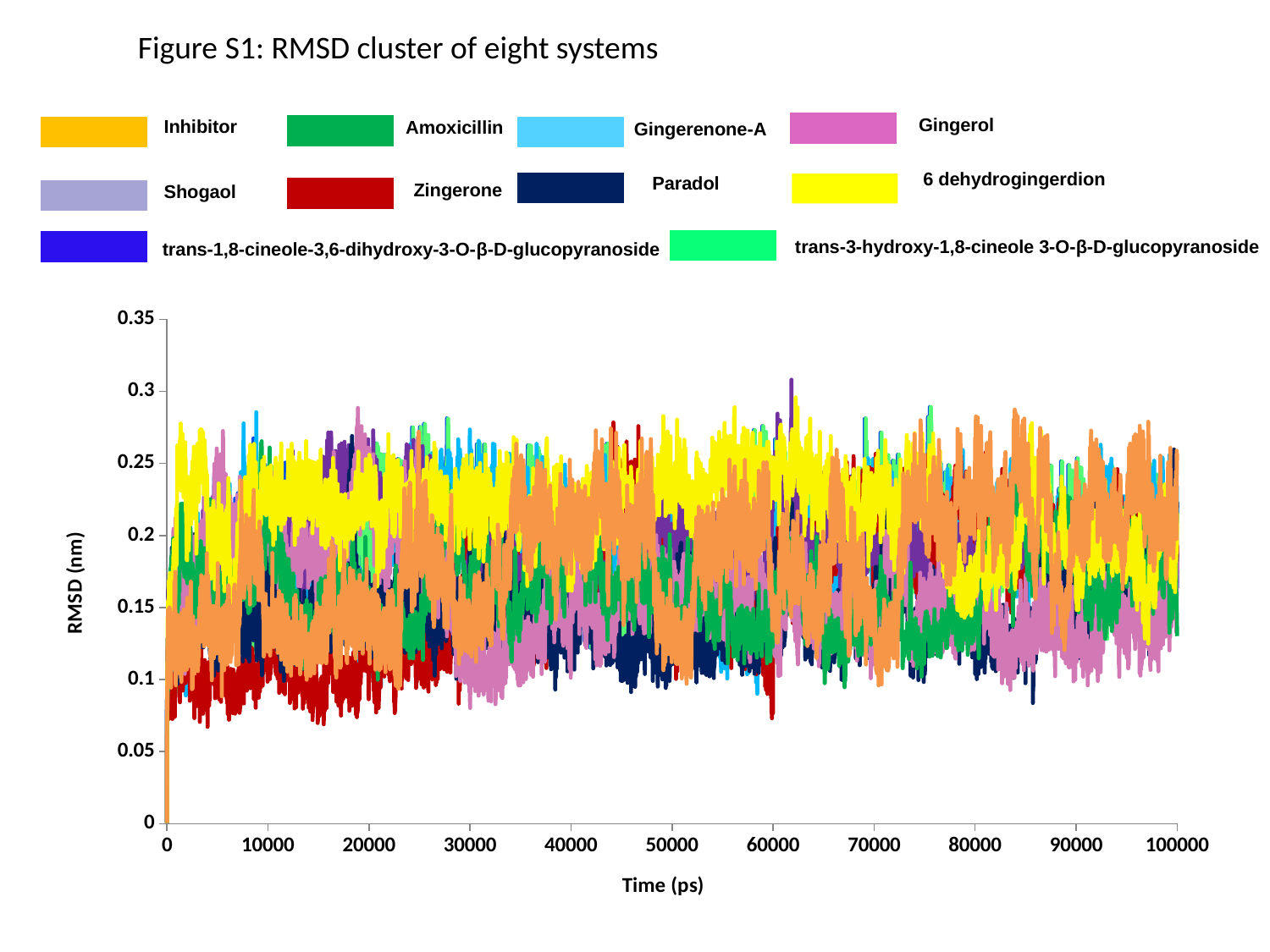

Figure S1: RMSD cluster of eight systems
Gingerol
Inhibitor
Amoxicillin
Gingerenone-A
6 dehydrogingerdion
Paradol
Zingerone
Shogaol
trans-3-hydroxy-1,8-cineole 3-O-β-D-glucopyranoside
trans-1,8-cineole-3,6-dihydroxy-3-O-β-D-glucopyranoside
### Chart
| Category | | | | | | | | | | | | | |
|---|---|---|---|---|---|---|---|---|---|---|---|---|---|

## Slide 4
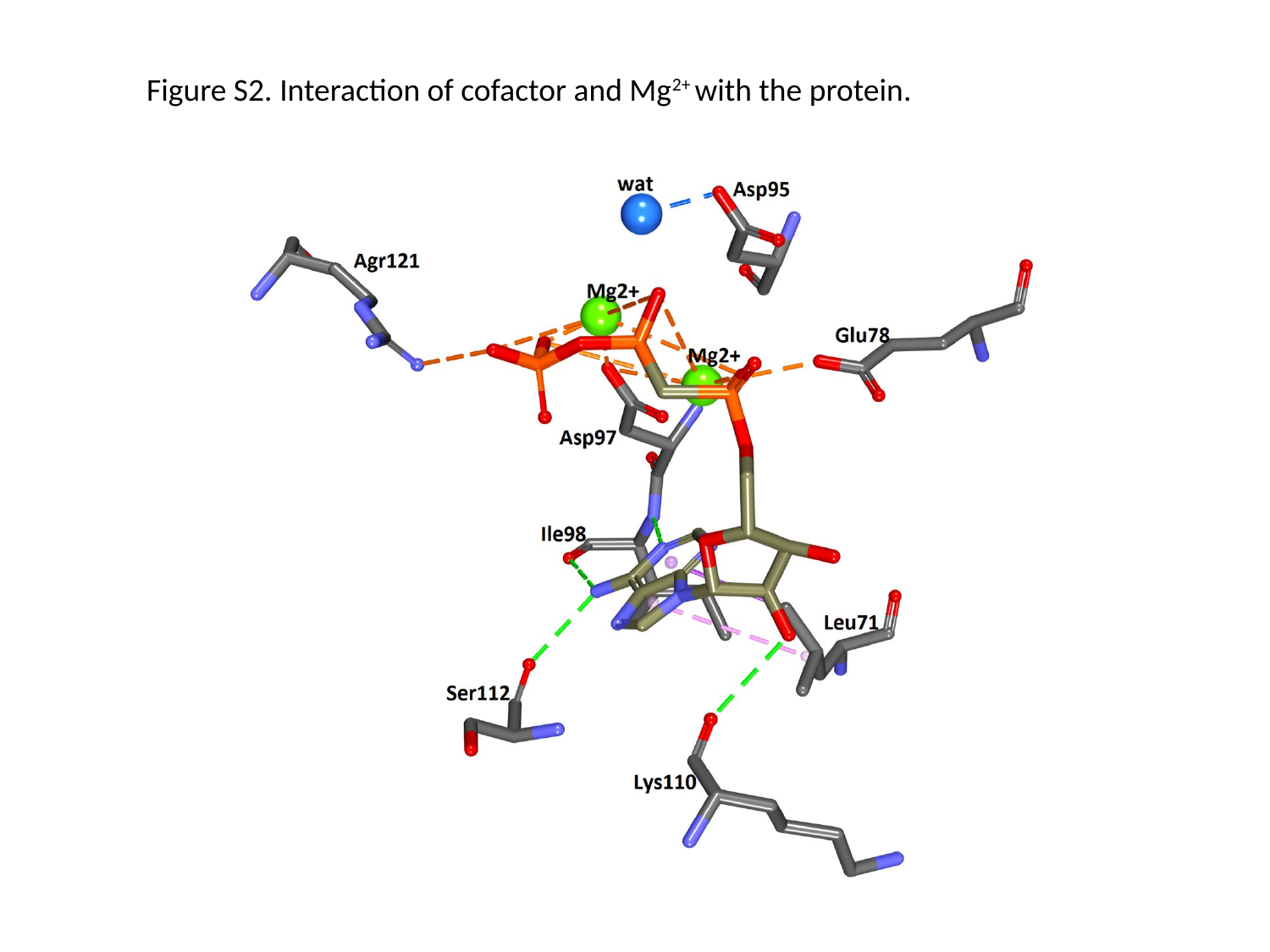

Figure S2. Interaction of cofactor and Mg2+ with the protein.
